# Supplementary material for: GluK1 kainate receptors are necessary for functional maturation of parvalbumin interneurons regulating amygdala circuit function
Source: Mol Psychiatry. 2024 Jun 28;29(12):3752–68. doi: 10.1038/s41380-024-02641-2 (PMC11609095; doi:10.1038/s41380-024-02641-2)
Supplement: Supplementary file 1 — Supplemental material [file 41380_2024_2641_MOESM1_ESM.pdf]

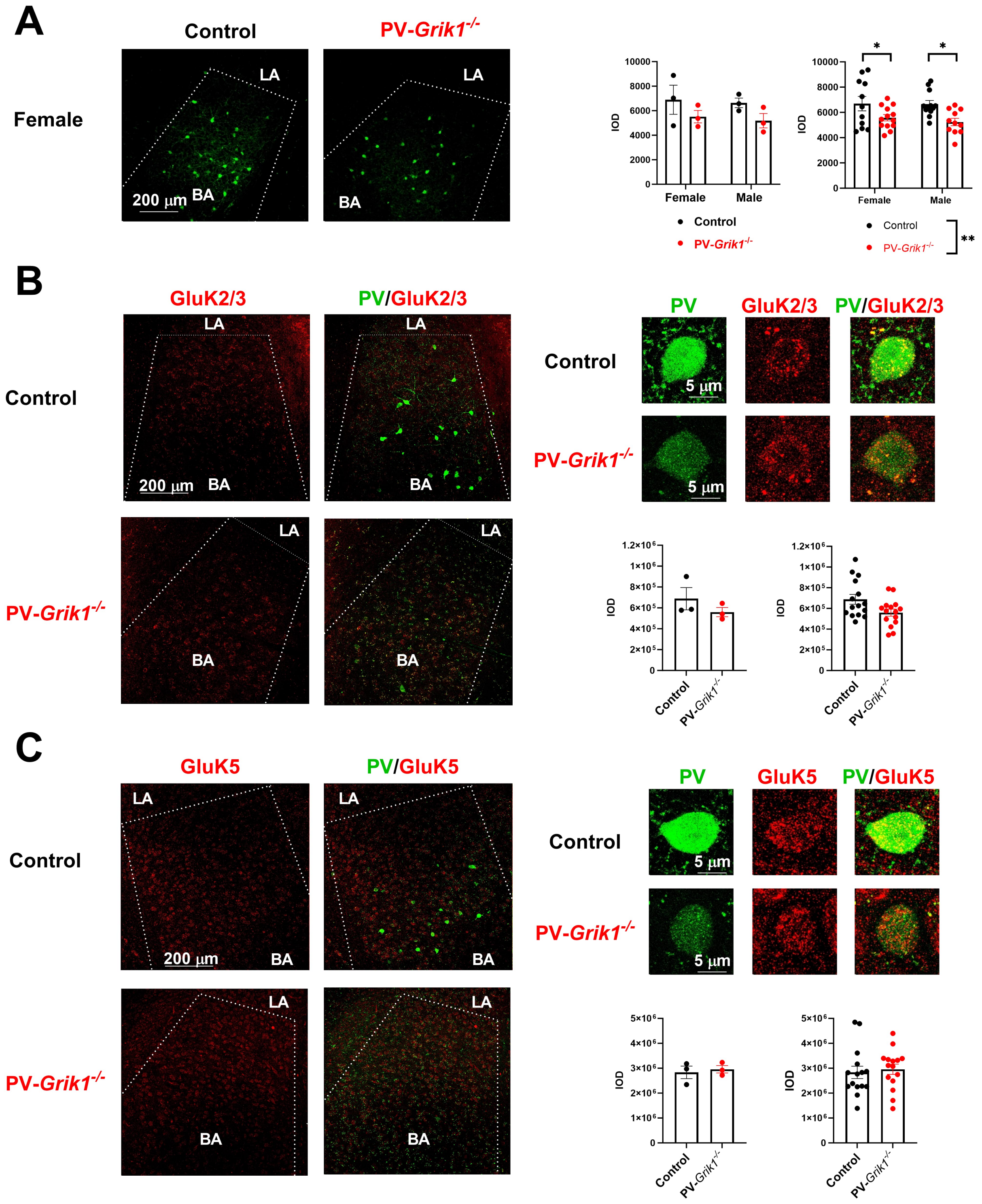

Supplementary Figure 1. Effect of GluK1 ablation on PV, GluK2/3 and GluK5 immunostaining in the BLA.

A. Example images of PV immunostaining in the BLA of adult female control and PV-*Grik1*$^{-/-}$ mice that are heterozygous for *Cre*. On the right, quantification of the IOD and PV+ cell densities in both adult female and male BLA. The data consist of 3 animals per group and 3 - 5 slices per animal and are shown for both, data averaged per animal (left) (genotype effect, IOD: $F (1, 9) = 4.542$, $p=0.0619$; 2-way ANOVA) and per section (genotype effect, IOD: $F(1, 43) = 12.27$, $p=0.0011$;* $p<0.05$, 2-way ANOVA with Holm Sidak).
B. Example images of GluK2/3 (red) and PV (green) immunostaining in the BLA of adult control and PV-*Grik1*$^{-/-}$ mice. On the right, example images of single PV+ GluK2/3+ cells as well as quantification of IOD of GluK2/3 in the PV+ cells both genotypes. The data consists of 3 animals per group and 5 slices per animal.
C. Similar data as in C, for PV (green) and GluK5 (red) immunostaining in the BLA of adult control and PV-*Grik1*$^{-/-}$ mice. The data consists of 3 animals per group and 5 slices per animal.

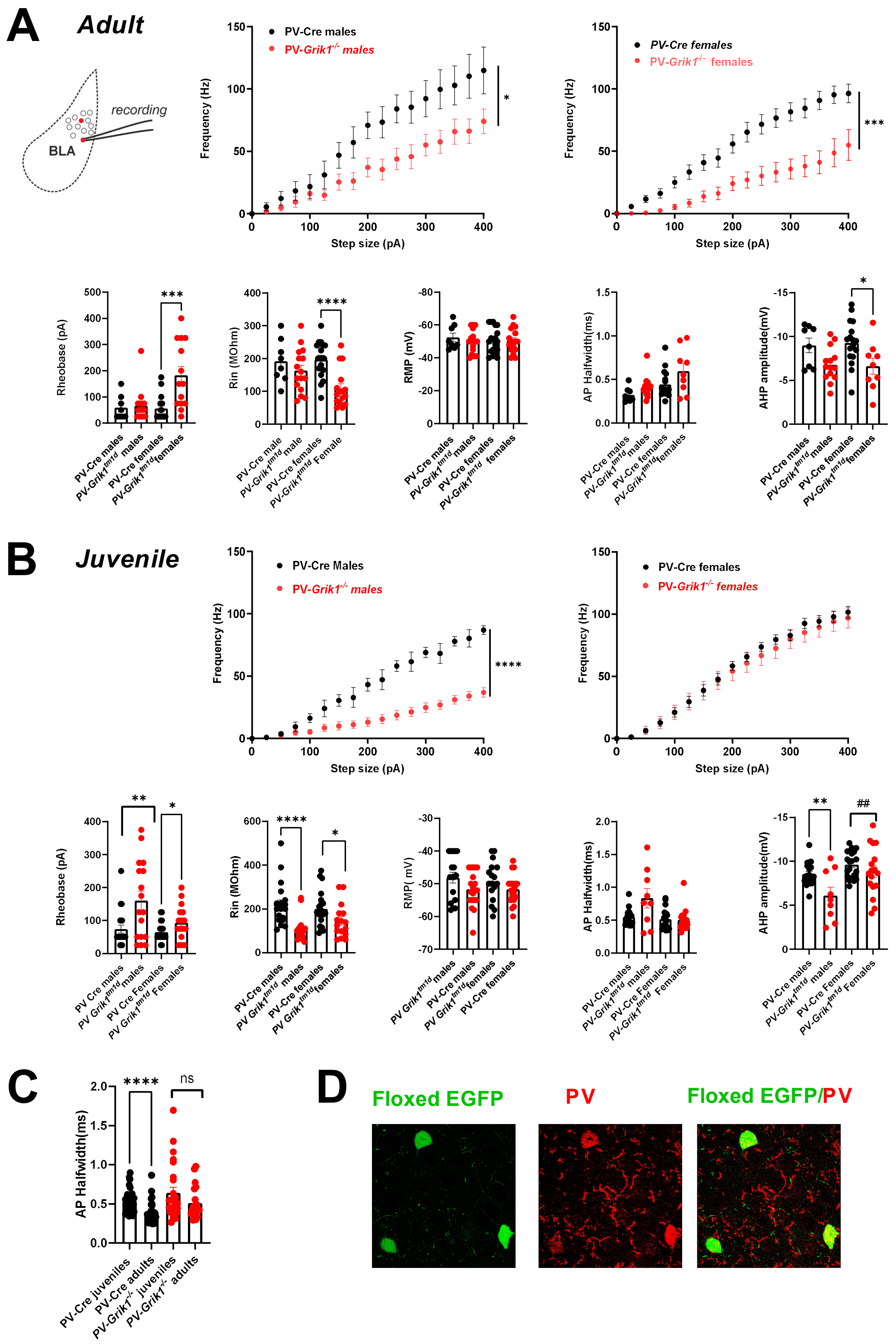

Supplementary Figure 2. Absence of GluK1 results in low PV+ cell excitability in both males and females across development.

A. PV IN excitability data from adult, from the same recordings as in Figure 3A, analysed separately for males and females. Control males: n= 8 cells, 3 animals, females: n=18 cells, 5 animals; PV-Grik1-/- males: n=15 cells, 3 animals, females: n= 11 cells, 4 animals. * $p < 0.05$; ****$p < 0.0001$; mixed effects model. Quantification of rheobase, input resistance, resting membrane potential (RMP), AP halfwidth and AHP amplitude. * $p < 0.05$; ***$p < 0.001$; **** $p < 0.0001$; two-tailed t-test and Mann-Whitney tests .

B. Similar data from juveniles, from the same recordings as in Figure 3 G. Control males: n = 20 cells, 4 animals, females: n=21 cells, 4 animals; PV-Grik1-/- males: n= 13 cells, 4 animals, females: n=17 cells, 4 animals. *$p < 0.05$; **$p < 0.01$; **** $p < 0.0001$; ## $p < 0.01$; f-test for variances

C. Data comparing AP halfwidth between the age groups, for the same data as shown in A and B. **** $p < 0.0001$ Mann-Whitney test .

D. Example images illustrating expression of virally delivered Cre-driven EGFP ('floxed EGFP') in PV-expressing cells in the BLA of PV-CRE mice, 14 days after injection of AAV viral particles into the neonate (P3) amygdala. Example images of illustrate co-localization of virally delivered EGFP and PV immunostaining (red), confirming specificity and efficacy of the neonatal transduction.

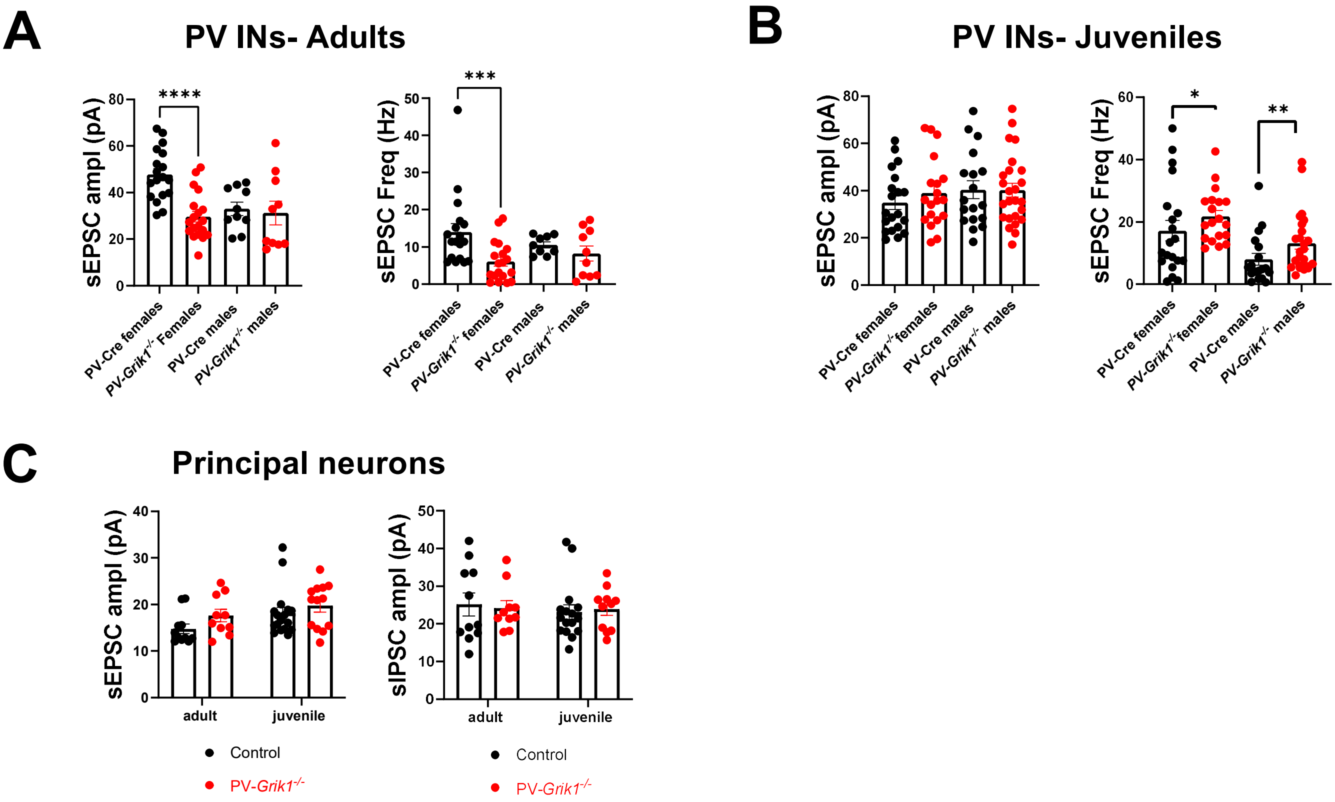

Supplementary Figure 3. Additional data related to analysis of spontaneous synaptic events in BLA PV INs (Figure 4) and principal cells (Figure 5B) in control and PV-Grik1-/- mice.

A. sEPSC frequency and amplitude data for the same adult PV IN recordings as shown in Figure 4A, analysed separately for males and females. Control females: n=18 cells 5 animals; males: n= 10 cells, 3 animals; PV-Grik1-/- females: n=19 cells, 4 animals; males: n=10 cells, 3 animals. ***p < 0.001; ****p < 0.0001; two-tailed t tests and Mann-Whitney test.

B. sEPSC frequency and amplitude data for the same juvenile PV IN recordings as shown in Figure 4B, analysed separately for males and females. Control males: n= 18 cells, 5 animals; females n= 20 cells, 4 animals; PV-Grik1-/- males: n=25 cells, 6 animals; females: n=19 cells, 4 animals.

C. sEPSC and sIPSC amplitude data for the same BLA principal neuron recordings that are shown in Figure 5B. Controls, Juvenile: n = 17 cells, 4 animals; adult: 11 cells, 5 animals; PV-Grik1-/- juvenile: n = 13 cells, 4 animals; adult: 10 cells, 5 animals.

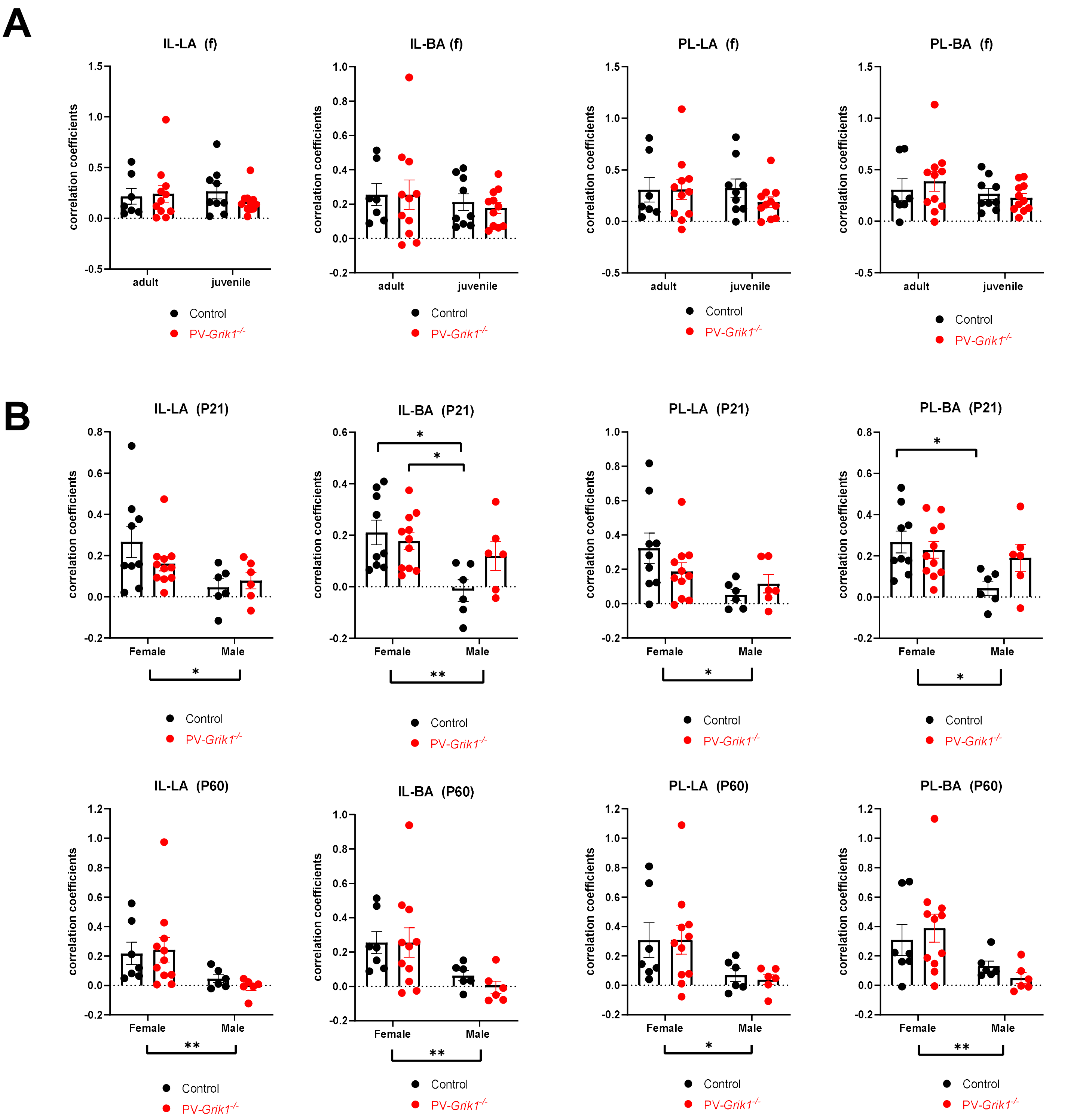

Supplementary Figure 4. Absence of GluK1 from PV INs results in age and sex specific changes in mPFC-BLA resting state functional connectivity.

A. fUS imaging data on resting state functional connectivity in adult (P60) and juvenile (P21) female control (n = 9) and PV-Grik1-/- (n = 11) mice. * $p < 0.05$, 2-way ANOVA. Similar data for males is shown in the Figure 6C.

B. Sex comparisons of resting state functional connectivity in juvenile (top row) and adult (bottom row) control and PV-Grik1-/- mice. The graphs are replotted from the same data as shown in Figure 6C and Supplementary Figure 4A. ** $p < 0.01$, *$p < 0.05$, 2-way ANOVA and Holm-Sidak. Values on the Y-axis indicate Pearson's correlation coefficients.
